# A new era of open access publishing in eating disorders

**DOI:** 10.1186/2050-2974-1-1

**Published:** 2013-01-22

**Authors:** Stephen Touyz, Phillipa J Hay

**Affiliations:** 1 School of Psychology and Centre for Eating and Dieting Disorders, University of Sydney, Sydney, Australia; 2 Schools of Medicine, Centre for Health Research University of Western Sydney and James Cook University, Queensland, Australia

## Background

We would like to welcome you to the first edition of the *Journal of Eating Disorders*. We are sure that the first questions you may wish to ask would be why a new journal of eating disorders and why an open access one? There can be no doubt that the field of eating disorders is experiencing a phase of major growth. We have strong international eating disorders organisations such as the Academy of Eating Disorders and the Eating Disorders Research Society and many national societies throughout the world. These societies not only provide local networks for their members but arrange annual conferences to ensure that important new developments in our field are rapidly disseminated. To meet the growing demand for the ever increasing research and reviews of developments in our field, the *International Journal of Eating Disorders* came into being in 1981. Under the excellent stewardship of the founding editor Craig Johnson and later Michael Strober the *International Journal* not only met the need for a home for publications in eating disorders but stimulated further interest that has exceeded every expectation.

Despite the explosion of knowledge emanating out of years of high calibre research in eating disorders, there remain many gaps. Whilst there is now sufficient scientific evidence supporting the effectiveness of cognitive behaviour therapy for the treatment of bulimia nervosa [1], further research needs to be undertaken to refine existing treatments to make them even more potent and cost-effective. Furthermore we know that such treatment protocols do not work for all such patients and there is an urgent need to develop-new ones or adapt existing protocols for those unable to respond and test treatments for other eating disorders. We have made significant progress in treating adolescents who have been ill for less than 3.5 years and Maudsley family-based treatment has rapidly become the treatment of choice for adolescents and children with anorexia nervosa [2]. However, although the data from randomised controlled trials has produced encouraging findings there is still little to guide clinicians as to the best approach in adults. In addition, there is almost no evidence to inform clinicians as to how best to proceed with a patient who has a more severe and enduring form of anorexia nervosa [3,4]. There are many important and exciting studies underway and the data emanating from these studies are likely to inform clinicians in our field for decades to come. New technologies such as functional MRI [5] and the burgeoning field of epigenetics [6] are also going to challenge our existing models of thinking about eating disorders and force us to reconceptualise aspects of what and how we deliver existing treatments. The digital revolution and the Internet in particular have provided opportunities that could only have been dreamt about only a few short years ago. The demands for collaborative research across not only countries but continents to deliver better research outcomes and the need for more rapid dissemination of outcomes cannot be overstated.

The time has now arrived to harness these opportunities and the *Journal of Eating Disorders* is uniquely placed to facilitate rapid publication and dissemination of the results of these exciting developments. The way we communicate our scientific findings is undergoing a metamorphosis and science has to adapt to meet these challenges. We have undoubtedly “crossed the Rubicon” and science as an open enterprise is now upon us [7]. The Netherlands has endorsed it and the Royal Society in the United Kingdom has become a leading advocate of open access publication. Pre-eminent research bodies such as the NIH [8] in the United States of America, the Wellcome Trust in the UK [9], and the National Medical Research Council in Australia have mandated that any research emanating out of their research grant funding has to be published either in an open access journal or one that will make the article available to all within a year.

The *Journal of Eating Disorders* aims to embrace and exploit all the opportunities that open access can deliver [10]. Adding to this its strong global commitment reflected in the nationalities of the Editorial Board. The Editors intend to rapidly respond to important developments in the field by also initiating special issues and then publishing these upon acceptance following peer review. Online open access, unlike the more traditional journals is not bound by page limitations. We are able to continue to publish our regular articles on acceptance whilst incorporating these special issues and invited papers. Once published such articles will be eventually be included in databases such as PubMed [11].

It is also important to understand the ramifications of open access. No longer is your research a private enterprise and read only by those who have access or the means to purchase your article, but by all who wish to enrich their knowledge. Clinicians, politicians, media, high school students, parents, teachers, health economists, private industry and those individuals living in developing countries [12] who do not have the means to purchase such articles, will now have access at the push of a Google button. Because of this open and rapid dissemination of research data, articles in open access journals such as the *Journal of Eating Disorders* are widely cited [13].

Notwithstanding all these advantages of open access over more traditional publications, there is a cost that has to be met. Producing a journal requires a production team to maintain databases, ensure high standards of formatting, marketing, PubMed listings and constantly upgrade our web page to name just a few. Upon acceptance of a paper and just prior to publication, the author is required to pay an Article Processing Charge [14,15]. Most universities are currently reviewing their policies as how best to support their academic and research staff to meet these costs whilst casting a careful eye on the ever increasing financial burden placed on shrinking budgets by the subscriptions required to maintain journal collections in libraries. It is also important to remember that unlike traditional journals, you the author retain the intellectual property of your own work and you do not assign it over to the journal. You therefore do not need to request permission from the journal when you wish to reprint sections of your work.

The Editors have deliberately kept the scope of the Journal broad. We invite not only original research reports but systematic reviews of the literature, clinical case reports, new randomised controlled trials, editorials as well as letters to the editors. We have a policy in place to provide a rapid turn around in review with the intention of publishing a paper within 8 weeks of submission. This is a tall order but one the Editors are determined to enshrine as one of the hallmarks of this new publication.

The launch contains papers reflecting the breadth diversity and scope of this Journal. Latner *et al.* examined the relationship between internalized weight bias and physical and mental health related quality of life (HRQoL) in 120 participants overweight or obese [16]. They found internalized weight bias was associated with greater impairment in HRQoL over and above the contributions of body mass index (BMI), age and medical comorbidities. Wade *et al.* investigated the role of perfectionism in body dissatisfaction in a large (over 1000) sample of adult women [17]. Controlling for BMI, a lower desired BMI was associated with higher levels of concern over mistakes and organization and a smaller ideal silhouette was associated with higher levels of concern over mistakes and doubts about action and organization. Finally Soh and Walter [18] in their systematic review point to the urgent need for more research in eating disorders from other cultures. This is an area in which we anticipate the *Journal of Eating Disorders* to lead the field.

On behalf of the Editors, Associate Editors, members of the Editorial Board as well as our superb management team, welcome to the *Journal of Eating Disorders* and we look forward to receiving your manuscripts for publication. It is our aim to provide you with a high quality journal which will be a flagship for the area in open access. Throughout this endeavour we have had the unwavering support of Sara Ho of BMC who never lost sight of our determination to launch a new open access journal in early 2013. Her encyclopaedic knowledge regarding the establishment of an exciting, innovative and new open access journal and all the details that needed to be worked through has ensured that our new journal of eating disorders has met the highest international standards. It is now there for all to see. We thank our managing editor Jeremy Freeman whose visionary talents, abundance of enthusiasm and attention to detail has contributed greatly to the quality of this edition but will ensure that we strive for bigger and better things in the years to come. A special word of thanks is to the Butterfly Foundation for their generous support to ensure that this dream was finally able to become a reality. We thank all who have contributed to this launch, the first important step in the journal’s role in facilitating the speed of quality science and practice in the field of eating disorder and related disciplines.

## Competing interests

The authors declare that they have no competing interests.

## Authors’ contributions

ST wrote the paper, PH contributed to content and editing. Both authors read and approved the final manuscript" should be present at the end of the paragraph.

## Authors’ information

Co-editors in Chief: Stephen Touyz and Phillipa Hay.
